# Outbreak of *Salmonella* Newport associated with internationally distributed raw goats' milk cheese, France, 2018

**DOI:** 10.1017/S0950268820000904

**Published:** 2020-05-04

**Authors:** E. Robinson, M. Travanut, L. Fabre, S. Larréché, L. Ramelli, L. Pascal, A. Guinard, N. Vincent, C. Calba, L. Meurice, MA. Le Thien, E. Fourgere, G. Jones, N. Fournet, A. Smith-Palmer, D. Brown, S. Le Hello, M. Pardos de la Gandara, FX. Weill, N. Jourdan-Da Silva

**Affiliations:** 1 Santé Publique France, Saint Maurice, France; 2 European Programme for Intervention Epidemiology Training (EPIET), European Centre for Disease Prevention and Control (ECDC), Solna, Sweden; 3 Agence Régional de Santé, Provence-Alpes-Côte d'Azur, Marseille, France; 4 French National Reference Center for Escherichia coli, Shigella and Salmonella, Institut Pasteur, Paris, France; 5 Mission des Urgences Sanitaires, Direction générale de l'alimentation, Paris, France; 6 Santé Publique France, Provence-Alpes-Côte d'Azur, Marseille, France; 7 Santé Publique France, Occitanie, Toulouse, France; 8 Santé Publique France, Ile de France, Paris, France; 9 Santé Publique France, Nouvelle-Aquitaine, Bordeaux, France; 10 Santé Publique France, Auvergne-Rhône-Alpes, Lyon, France; 11 Health Protection Scotland, Glasgow, UK; 12 Scottish Microbiology Reference Laboratories, Glasgow, UK

**Keywords:** Outbreaks, raw milk collection, *Salmonella*, surveillance

## Abstract

Raw milk cheeses are commonly consumed in France and are also a common source of foodborne outbreaks (FBOs). Both an FBO surveillance system and a laboratory-based surveillance system aim to detect *Salmonella* outbreaks. In early August 2018, five familial FBOs due to *Salmonella* spp. were reported to a regional health authority. Investigation identified common exposure to a raw goats' milk cheese, from which *Salmonella* spp. were also isolated, leading to an international product recall. Three weeks later, on 22 August, a national increase in *Salmonella* Newport ST118 was detected through laboratory surveillance. Concomitantly isolates from the earlier familial clusters were confirmed as *S.* Newport ST118. Interviews with a selection of the laboratory-identified cases revealed exposure to the same cheese, including exposure to batches not included in the previous recall, leading to an expansion of the recall. The outbreak affected 153 cases, including six cases in Scotland. *S*. Newport was detected in the cheese and in the milk of one of the producer's goats. The difference in the two alerts generated by this outbreak highlight the timeliness of the FBO system and the precision of the laboratory-based surveillance system. It is also a reminder of the risks associated with raw milk cheeses.

## Background

Raw milk cheeses are popular in France accounting for 15% of all mature French cheeses, 75% of protected designation of origin (PDO) and protected geographical indication (PGI) cheeses, and the majority of other artisanal cheeses [1]. In the most recent national nutritional survey, 32% of people reported having eaten a raw milk cheese in at least one of three non-consecutive days in the previous 3 weeks [2]. Collaterally, raw milk cheeses have been the vehicle of 16 major *Salmonella* foodborne outbreaks (FBO) in France between 2008 and 2018, representing a third of *Salmonella* outbreaks with an identified source [3–7].

There are two French surveillance systems with the objective of detecting *Salmonella* FBOs. The syndromic FBO surveillance system is based on mandatory notification to regional health agencies of clusters of gastrointestinal illness involving at least two cases having shared a common meal, and whose cause is therefore suspected to be foodborne. Once validated as true clusters of illness, they are notified to the national public health agency, Santé publique France (SpF). In 2016, 1455 clusters involving 13 997 ill persons were notified [8]. *Salmonella* spp. is the most frequent causal agent amongst clusters with an identified agent, responsible for 41% of such clusters between 2007 and 2016 [9]. In around half of salmonellosis clusters, the serotype is not confirmed.

The second surveillance system is laboratory based. The French national reference centre for *Escherichia coli*, *Shigella* and *Salmonella* (FNRC-ESS) located at Institut Pasteur receives isolates from a voluntary network of laboratories across France. It was previously estimated that the laboratory surveillance system captured 66% of laboratory-confirmed human *Salmonella* infections in France [10]. On a weekly basis, algorithms are applied to FNRC-ESS data to detect unusual increases by serotype.

*Salmonella enterica* serotype Newport (*S*. Newport) is an uncommon cause of sporadic salmonellosis and *Salmonella* outbreaks in France. It accounts for 1–2% of human cases per year and since 2000 has been the responsible agent in five FBOs – four linked to different raw goats' milk cheeses and one linked to horsemeat [10, 11]. We describe a fifth outbreak of *S.* Newport linked to a raw goats' milk cheese which was detected separately by both of the French surveillance systems.

## Outbreak detection

### The first alert

Between 2 and 8 August 2018, five familial clusters of gastrointestinal illness, originating from one geographic department (department 13 – Bouches-du-Rhône), were reported to the regional health agency of Provence-Alpes-Côte d'Azur (PACA) in the south of France as part of the FBO surveillance system. These five clusters included 15 ill persons with dates of symptom onset between 28 July and 8 August 2018. Stool samples from nine of 10 tested cases were positive for *Salmonella* spp. and isolates were sent to the FNRC-ESS for typing. Investigation by the regional health agency identified that the consumption of a particular raw goats' milk cheese manufactured by one French producer (X) was common to all clusters. Samples of two batches of the cheese from a vendor common to two of the clusters and samples of two further batches from the producer were positive for *Salmonella* spp. A product recall was issued on 10 August 2018 [12]. This recall concerned cheeses produced since 10 July 2018 and placed on the market since 21 July 2018. As the product had been distributed internationally, a notification (2018.2296) was issued on 13 August through the Rapid Alert System for Food and Feed (RASFF), a European Commission platform to rapidly exchange information when risks to public health are detected in the food chain [13].

### The second alert

On 22 August 2018, a national increase in *S.* Newport multilocus sequence type 118 (*S*. Newport ST118) cases was detected through the FNRC-ESS weekly algorithms. As of 22 August, the FNRC-ESS had detected 36 isolates of *S.* Newport ST118 with a sampling date between 10 July and 2 August. This compared with a total of eight isolates in July and August of 2017. Two of the isolates (detected on 21 August) belonged to cases associated with the previously described familial clusters.

We hypothesised that the national increase in *S*. Newport ST118 cases was associated with the same cheese. An investigation was conducted to confirm the hypothesis, evaluate the extent of the outbreak and determine if further control measures were needed.

## Methods

### Case definitions

We defined a confirmed case as a person with a laboratory-confirmed *S*. Newport ST118 isolate belonging to the outbreak cluster on phylogenetic analysis (i.e. core genome MLST (cgMLST) hierarchical clustering of five or less alleles or <5 single nucleotide polymorphisms (SNPs) to another isolate) in a sample taken after 1 July 2018. We defined a probable case as a person with an epidemiological link to a confirmed case and either a non-serotyped *Salmonella* spp. isolate in a sample taken after 1 July or the onset of gastrointestinal illness after 1 July. This probable case definition was chosen in order to be inclusive of members of clusters where investigative protocols do not require testing or serotype confirmation of all cases. We defined a possible case as a person with a laboratory-confirmed *S*. Newport isolate in a sample taken after 1 July for whom cluster analysis was not possible and epidemiological links were not known.

### Epidemiological investigations

Amongst the 36 cases of *S*. Newport ST118 detected by the FNRC-ESS between 1 July and 22 August, 25 (who were not known to be related to the previously investigated clusters) were selected to be interviewed using the cheese component of the national salmonellosis trawling questionnaire. Interviewers were instructed not to probe about the suspected cheese unless consumption was disclosed by the case. Cases were chosen to reflect different geographic regions and sampling dates. Seven of the cases selected had sampling dates which would correspond with illness and potential exposure prior to 21 July (the date the previously recalled products were placed on the market). These were selected to accurately date the outbreak onset and determine if earlier product batches may also have been implicated and thus require an extension of the recall.

Given the international distribution of the product, European public health authorities were alerted through the European Epidemic Intelligence Information System (EPIS), operated by the European Centre for Disease Prevention and Control (ECDC), on 24 August in order to identify cases outside of France. Sequencing data of the outbreak strain was shared when it became available.

### Human microbiological investigations

Whole genome sequencing (WGS) was performed as part of routine procedures at the FNRC-ESS. WGS was carried out at the Plateforme de microbiologie mutualisée (P2M) from the Pasteur International Bioresources network (PIBnet, Institut Pasteur, Paris, France). The MagNAPure 96 system (Roche Diagnostics, Indianapolis, IN, USA) was used for DNA extraction, libraries were prepared using the Nextera XT kit (Illumina, San Diego, CA, USA) and sequencing was done with the NextSeq 500 system (Illumina). Serotype prediction was done by in-house scripts based on MLST [14], *fliC* and *fljB* gene databases (FNRC-ESS internal flagellin database, unpublished). All genomic sequences (*n* = 209) available from *S*. Newport ST118 isolates received at the FNRC-ESS between 1 January 2015 and 20 October 2018 were deposited into EnteroBase (https://enterobase.warwick.ac.uk) [15]. Phylogenetic analysis was performed by two different approaches integrated into EnteroBase: SNP analysis and cgMLST hierarchical clustering, by the ‘Legacy cgMLST v2 + HierCC’ tool in August 2018, and confirmed in August 2019 for this publication by the new tool ‘cgMLST V2 + HierCC V1’. Raw reads of a representative outbreak isolate (201805061) have also been deposited to the European Nucleotide Archive (ENA) (http://www.ebi.ac.uk/ena), under study accession number PRJEB29425.

### Product and environmental investigations

As part of the investigation of the first alert, two batches of the cheese (produced on 10 July and 19 July) which were still available at a vendor which supplied two of the clusters, were sampled by the population protection directorate (DDPP) of department 13. The environmental health authority where Producer X was located also undertook an investigation which included environmental sampling and microbiological testing of the production facility, product samples, bulk-milk samples and milk samples from each of the supplying animals. Serotyping of positive samples was undertaken by the French Agency for Food, Environmental and Occupational Health & Safety (ANSES). The French Directorate General for Food (DGAL) undertook traceback and traceforward investigations.

## Results

### Descriptive epidemiology

**Fig. 1.**
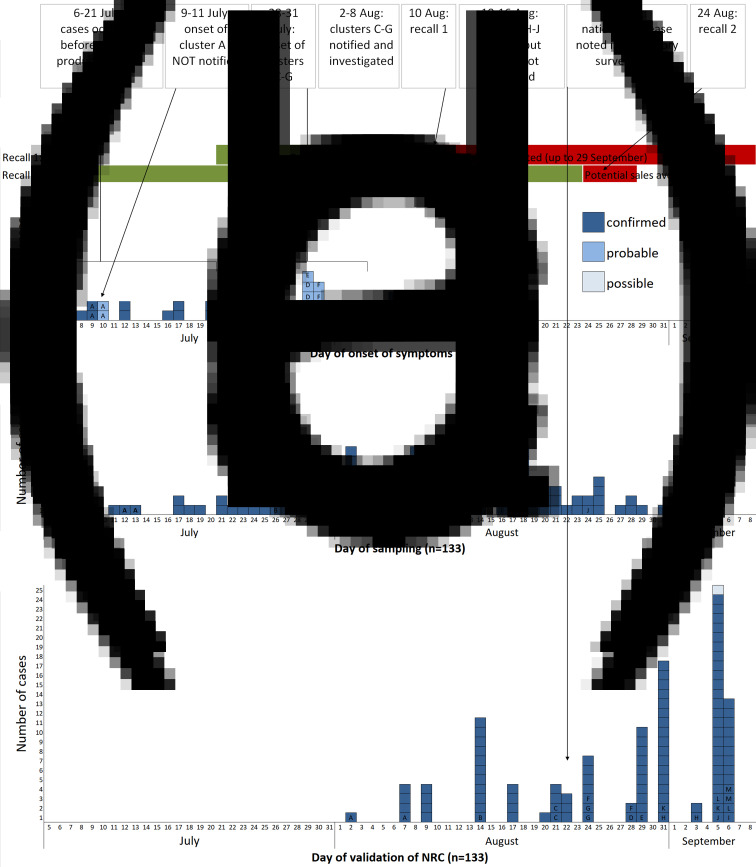
(a) Chronological order of outbreak events; (b) chronology of the distribution and recall of implicated products; (c) epicurve of *S*. Newport ST118 cases by day of onset of symptoms, France, 2018 (*n* = 37; letter indicates cluster case belongs to); (d) epicurve of *S*. Newport ST118 cases by day of sampling, France, 2018 (*n* = 133, one late case with sampling date of 27 September not shown); (e) epicurve of *S*. Newport ST118 cases by date of validation of serotype by the FNRC-ESS, France, 2018 (*n* = 109, cases with date of validation after September 7 (*n* = 25) not shown).

Within France, 147 cases (133 confirmed, 13 probable, 1 possible) were identified. All nine cases from the familial clusters of the first alert (identified through the FBO surveillance system) with isolates available were confirmed. Date of sampling was between 11 July and 27 September, and peaked during week 32 (August 6–7; Fig. 1d). When known (*n* = 37), symptom onset was between 6 July and 8 August, and peaked during week 30. Fifty-two per cent (74/142) of cases were male and the median age was 46 years (range: 3–87 years). Of 38 cases whose clinical history was known, 13 were hospitalised. One case died, but salmonellosis was not the principal cause of death and information on food exposure was not available.

Between 24 and 25 August, 20 cases were interviewed with 15 reporting definite or likely consumption of the suspected goats' cheese prior to symptom onset. Two cases consumed the cheese of Producer X, which had been purchased directly from the farm, at the same family event on 8 July, before the production of the cheeses included in the first recall. Two other attendees of the event were also ill (probable cases). This familial foodborne cluster (cluster A) was not notified to the regional health agency. Another case had consumed the cheese of Producer X after purchasing it on 15 July, again before products involved in the first recall had been placed on the market.

Table 1 summarises the clusters within the outbreak. All of the five familial clusters (C–G) reported in department 13 had at least one confirmed case. Three other familial clusters (H–J) from different departments, which were initially notified on 10, 16 and 24 of August were also found to be part of the outbreak, although the source was not initially identified as the implicated cheese. Interviews with cases identified two other familial clusters which had not been notified. The attack rate amongst the known clusters ranged between 29% and 100%.

**Table 1.** Summary of clusters that were part of the outbreak

Cluster ID	Confirmed cases	Probable cases	Attack rate (%)	Dates of symptom onset	Notified	Date of notification	Region notifying	Suspected source at the time of notification
A^a^	2	2	Nk	9–10 July 2018	No	Na	Na	Na
B^a^	1	2	Nk	20–26 July 2017	No	Na	Na	Na
C	2	0	50	28 July 2018	Yes	6 August 2018	PACA	Implicate cheese
D	1	2	75	29 July 2018	Yes	2 August 2018	PACA	Implicate cheese
E	1	1	29	29 July 2018	Yes	8 August 2018	PACA	Implicate cheese
F	2	4	50	30 July 2018	Yes	6 August 2018	PACA	Implicate cheese
G	2	0	50	31 July 2018	Yes	6 August 2018	PACA	Implicate cheese
H	2	1	100	6 August 2018–8 August 2018	Yes	10 August 2018	Occitanie	Meal of omelette, unspecified cheese and salad
I	2	0	100	11 August 2018–16 August 2018	Yes	16 August 2018	Ile de France	Meal of different cheeses
J	1	1	100	Nk; date of sampling 24 August 2018	Yes	Nk	Ile de France	Meal of different cheeses

Nk, not known; Na, not applicable; PACA, Provence-Alpes-Côte d'Azur.

a Cluster identified during the investigation through case interview.

Through EPIS, Scottish authorities reported six cases of *S*. Newport ST118, with symptom onset between 20 July and 15 August, clustering within five SNPs on phylogenetic analysis undertaken by the Scottish Microbiology Reference Laboratories (SMiRL). Four of these cases reported consumption of the implicated cheese in Scotland where it was sold by a single vendor, one reported the consumption of a variety of unspecified cheeses while travelling in Spain during their exposure period, and for one, information on exposure to the product was not known. No other international cases were reported.

### Microbiological results

**Fig. 2.**
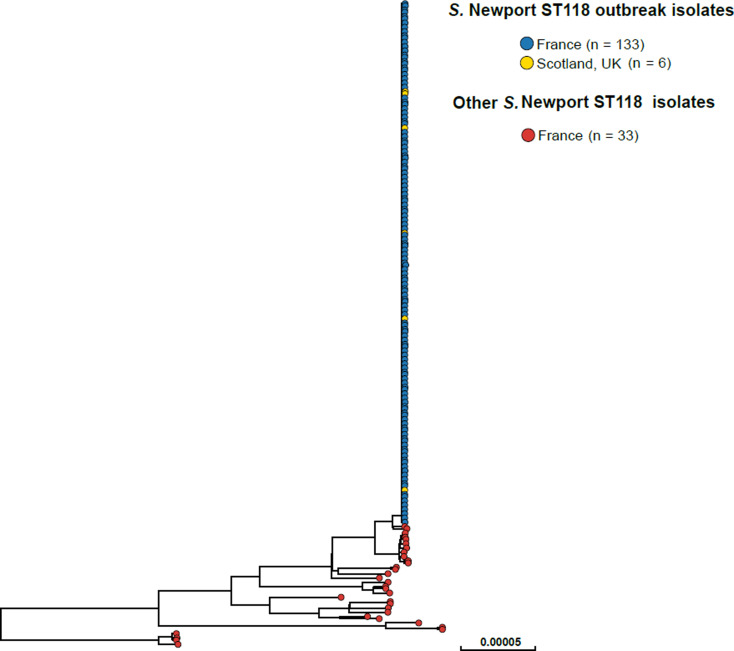
Phylogenetic tree of 172 *S.* Newport ST118 isolates analysed by WGS at the FNRC-ESS in France and the SMiRL in Scotland, UK, in 2018. The tree was made by using the ‘create SNP project’ tool in EnteroBase and was based on 5042 non-repetitive SNPs. The tips are coloured according to the country of isolation and outbreak status.

SNP analysis revealed that the majority of the 2018 French *S*. Newport ST118 human isolates (133/166, 80.1%) clustered tightly together (Fig. 2). These clustered isolates also displayed an identical type ‘133068’ by hierarchical clustering of cgMLST data differing by five or less alleles (‘HC5’). Other isolates on the SNP-based phylogenetic tree showed different cgMLSTHC5 cluster types (data not shown). The search for other *Salmonella* isolates displaying HC5|133068 cluster type in Enterobase (175 788 *Salmonella* genomic sequences by 28 October 2018) identified six other non-redundant human isolates submitted by the SMiRL, corresponding to the six cases previously reported by Scotland. These Scottish isolates also belonged to the same SNP cluster as the French outbreak isolates (data not shown). French outbreak isolates were pansusceptible.

### Environmental and product investigation results

The implicated cheese, an artisanal product with PDO status, was the only product of producer X, who only used milk from his own herd. A batch of approximately 800 units (each weighing around 60 g) was produced nearly every day. The cheese was distributed across France and internationally. In Europe, the product was distributed to Belgium, Germany, Italy, Luxembourg, Spain, Switzerland and the UK [13].

As previously mentioned, during the investigation of the first alert, four separate batches (sampled from a vendor and from the producer) produced between 10 July and 4 August had tested positive for *Salmonella* spp., leading to the first recall. On 24 August, during the investigation of the second alert, these isolates were confirmed as *S.* Newport (serotyping by agglutination). Given the epidemiological results and the rarity of the serotype, food isolates were not sequenced to further confirm the association. No cheese produced before 10 July was available for testing. Milk from one animal tested positive for *S.* Newport.

## Outbreak control measures

The cases detected by the FNRC-ESS that occurred prior to 21 July (date on which batches from first recall were placed on the market; Fig. 1a), and the confirmation that some cases had purchased the cheese before this date, suggested that earlier batches were also contaminated. A second recall was issued on 24 August which concerned batches produced since 24 June 2018 [16]. As no further product was placed on the market after the first recall and the shelf life was 2 months, this second recall effectively covered any remaining products potentially still on the market. Both recalls were accompanied by a national press release in addition to notices at vendors. The RASFF notification was updated to reflect the expanded recall.

Producer X had halted production at the time of the first alert pending investigation. The source animal was removed from the herd and the facility underwent rigorous cleaning. After resumption of production, Producer X was subject to enhanced microbiological monitoring and environmental and product samples remained negative for *Salmonella*.

## Discussion

We have described a large outbreak of pansusceptible *S*. Newport linked to an internationally distributed artisanal raw goats' milk cheese produced in France. The magnitude of the outbreak is likely underestimated. In France, a multiplication factor of 20 has been estimated between *Salmonella* cases ascertained by the laboratory surveillance system and cases in the community [17]. The high attack rate seen in the familial clusters, and that over 23 000 potentially contaminated units were distributed in France and internationally, further suggests an underestimation of the magnitude both in France and internationally. No international cases were reported except for those reported from Scotland. As only 24 units implicated in the first recall were sold in Scotland, and four cases known to have been exposed to these units were identified, it seems likely that cases also occurred in other countries that received the product. However, the first recall is likely to have mitigated against a much larger outbreak.

While five of six *S.* Newport outbreaks identified in France have now been associated with raw goats' milk cheeses, we are not aware of any particular affinity between this serotype and goats or dairy products. Elsewhere the sources in *S.* Newport outbreaks have been eclectic, including fresh produce (fruits, leafy, root and vine-stalk vegetables, sprouts, nuts) [18–24], ground beef [25, 26] and other animal-derived foods [27], suggesting varied reservoirs. Two other outbreaks linked to consumption of raw milk cheeses have been reported – both were in the USA and associated with unpasteurised cows' milk cheeses [28, 29]. Interestingly, in the USA, *S*. Newport isolates with a pansusceptible profile are often associated with fresh produce and potential environmental reservoirs while multidrug-resistant isolates are associated with animal products, leading to a hypothesis that pansusceptibility is an indicator of an environmental reservoir, or an environment with less antibiotic exposure [27, 30]. Contact with amphibians and reptiles has also been shown to be associated with pansusceptible infection, in keeping with the hypothesis of the association between pansusceptibility and environmental reservoirs [31]. Isolates in this outbreak were pansusceptible. To comply with the PDO requirements, the goats producing the implicated cheese must spend the majority of time outside and feed on natural vegetation and therefore may have been exposed to an environmental reservoir.

The outbreak again highlights the potential risks associated with raw milk products. Raw goats' milk can be contaminated by similar human gastrointestinal pathogens as raw cows' milk [32, 33]. In France, six other *Salmonella* outbreaks have been linked to French raw goats' milk cheeses and three Shiga toxin-producing *Escherichia coli* (STEC) outbreaks have been linked to mixed raw goats' and cows' milk cheeses [3, 4, 34, 35]. Gastrointestinal outbreaks of STEC, *Cryptosporidium parvum*, *Campylobacter* spp. and *Salmonella* spp. associated with consumption of raw goats' milk or its cheese have been reported by other countries [33, 36]. In addition, consumption has also been associated with other non-gastrointestinal zoonotic infections including tick borne encephalitis, brucellosis, toxoplasmosis, Q fever, *Streptococcus* equi subspecies zooepidemicus, Rift valley fever and tuberculosis [33].

Similarly to cows' milk, contamination of goats' milk can be due to direct shedding into the milk from the udder or external contamination during or after milking, depending on the pathogen. It is, however, thought that faecal contamination is less likely than for cows' milk as goats' faeces are pelletised and drier, therefore less likely to contaminate the udder. Also, compared to grazing cows, goats' udders are less likely to come into contact with faecally contaminated mud. In four of the seven documented *Salmonella* outbreaks in France associated with goats' milk cheese (including the current outbreak), one or more goats were found to be asymptomatic udder excretors, while in a fifth, a goat with clinical mastitis was found to be the source. In the STEC outbreaks associated with mixed raw milk, the pathogen was found in the faeces of one or more animals of both species or in the production facility, but not in excreted milk.

Consumption of goats' milk products is less common than that of cows' milk, although there may be geographic variations. In the European Union, goats' milk production accounted for only 1.4% of total milk production [37]. In the French nutritional survey, previously mentioned, 0.4% of people reported drinking goats' milk (2; personal communication, SpF). Fifteen per cent of people reported eating any type of goats' milk cheese, and 3% reported eating raw goats' milk cheese. This compares to 27% of people who reported eating raw cows' milk cheese. Raw goats' milk cheeses account for about 9% of French raw milk cheese produced. While outbreaks associated with raw goats' milk in France are less frequent than cows' milk, the number seems disproportionate given the differences in consumption. Of the 18 *Salmonella* outbreaks linked to raw milk cheeses in France between 2004 and 2018, six have been linked to a goats' milk cheese. In addition, raw goats' milk cheeses accounted for 26% of the 101 food safety alerts (mostly non-conformities in quality-controls by the producer) relating to raw milk cheeses notified to the DGAL [38]. The disproportionality in pathogens and disease associated with goats' milk cheeses may be because raw goats' milk cheeses are more likely to be soft cheeses, in which the high water content and pH permits survival and enhances growth conditions for pathogens [39, 40]. During 2018, the DGAL undertook a study which involved random sampling at production sites of raw cheeses from all species to determine the prevalence of *Listeria*, *Salmonella* and STEC in products. The results of this study will provide a clearer picture of the risk posed by raw cheeses from different species.

France produces half of the total EU production of cheese from pure goats' milk [37]. Twenty-two per cent of goats' milk produced in France is processed on the farm, mainly without pasteurisation, with 18–20 000 tonnes of artisanal cheeses produced each year. This outbreak highlights how even a relatively small-scale producer can contribute to a large-scale, international outbreak. A third of French goats' milk cheese is exported. The artisanal raw milk goats' cheese market is reportedly growing in other regions such as the UK and Australia. While newer non-thermal techniques to improve microbiological quality may be an option for industrial producers of raw milk cheeses, they are unlikely to be feasible for artisanal producers. Many such producers could not afford to invest in these technologies. More importantly, they are unlikely to be acceptable to the producer or to consumers, who are often keen to retain traditional production methods. Regulations around PDO/PGI products also require adherence to traditional production methods.

In European countries, raw cheese production and sale is subject to EU food safety regulations. While additional good practice guidelines have been developed in collaboration with industry in some regions [41], artisanal producers and their suppliers, in France and elsewhere, may need particular support to undertake the required risk assessments and implement critical control procedures to maximise the microbiological safety of their product. However, while improved husbandry and production practices in combination with microbiological testing protocols can mitigate against the contaminated product, the risk cannot be eliminated and consumers of all raw milk products should be aware of the potential risks, especially those at increased risk of severe illness. In June of 2019, the DGAL recommended that young children, particularly under 5 years, pregnant women and immunocompromised persons not consume raw milk cheeses [42].

The other interesting aspect of this outbreak is the time difference, of over 2 weeks, between its detection in the FBO syndromic surveillance system and the laboratory surveillance system, highlighting some of the strengths and weaknesses of both. The FBO surveillance system offers timeliness, as clusters should be reported before microbiological confirmation of cases. In 2016, 31% of clusters were reported on the day or the day after the onset of symptoms of the first ill person [8]. This prompt reporting can facilitate the investigation and rapid implementation of control measures. In addition, in the event of an alert originating from the laboratory system, a search for clusters of the same serotype within the FBO surveillance system is undertaken, and when present can aid hypothesis generation. A study (covering the period 1995–2000) estimated that 26% of salmonellosis clusters with microbiologically confirmed cases were captured by the FBO syndromic system [43]. In addition, linking reported clusters, particularly when they span multiple geographic regions, is limited by imprecise or absent information on the pathogen or suspected source. Three other familial *Salmonella* clusters within this outbreak, which were retrospectively microbiologically linked, were notified from different regions prior to the second alert. A meal containing cheese was the suspected vehicle for each but the implicated cheese was not specifically identified. Therefore, they were not linked to the clusters of department 13, and an opportunity for the earlier recognition of the true extent of the outbreak was missed. Similarly, linking clusters based on a common causative pathogen can be hindered by the fact that in around half of salmonellosis clusters, the serotype is not confirmed at the time of the notification [8].

In contrast, the laboratory-based surveillance offers greater sensitivity, with an estimated 66% of laboratory-confirmed infections captured [10]. It also provides detailed pathogen information, an advantage which has been further enhanced since the introduction of WGS. However, this comes at the cost of timeliness, with an average analysis delay (receipt in the FNRC-ESS to the validation of serotype) of 26 days compared to 6 days when serotyping was by agglutination.

Although the first recall stemming from the FBO surveillance system was inadequate as the extent of the outbreak and contamination was not fully evident, it still occurred almost 2 weeks before the outbreak was even detected through laboratory surveillance. Timeliness can be one of the limitations of the use of WGS for outbreak detection. Turnaround times will depend on demand and available resources and the high incidence of *Salmonella* can be one of the challenges to routine implementation of WGS in some countries [44]. However, delays will likely decrease in the future as methodological advances and reduced costs improve capacity. Having said that, the availability of WGS enabled timely international collaboration and the confirmation of international cases during this outbreak. This outbreak highlights the value of both surveillance systems. While still advancing towards and investing in WGS surveillance, public health authorities should continue to support such traditional systems within multifaceted surveillance programmes in order to optimise the timeliness and responsiveness of surveillance.

This result is also a reminder of the risks associated with raw milk cheeses and that measures to prevent the entry of pathogens into the artisanal raw milk cheese production chain and the optimisation of surveillance aimed at the prevention of FBO such as in France should be extended or strengthened in other regions of Europe with tradition in the production of cheeses made from raw milk.
